# 2-Amino-4-[4-(dimethyl­amino)­phen­yl]-5-oxo-5,6,7,8-tetra­hydro-4*H*-chromene-3-carbonitrile

**DOI:** 10.1107/S1600536811043662

**Published:** 2011-10-29

**Authors:** Yan Qiao, Guifang Chen, Lingqian Kong, Xiuping Ju, Zhiqing Gao

**Affiliations:** a Dongchang College, Liaocheng University, Liaocheng 250059, People’s Republic of China; b College of Chemistry and Chemical Engineering, Liaocheng University, Shandong 250059, People’s Republic of China

## Abstract

In the title mol­ecule, C_18_H_19_N_3_O_2_, the fused cyclo­hexenone and pyran rings adopt sofa conformations. Inter­molecular N—H⋯N and N—H⋯O hydrogen bonds link mol­ecules into corrugated layers parallel to the *bc* plane.

## Related literature

For the crystal structures of related compounds, see: Kong *et al.* (2011 ▶); Wang (2011 ▶).
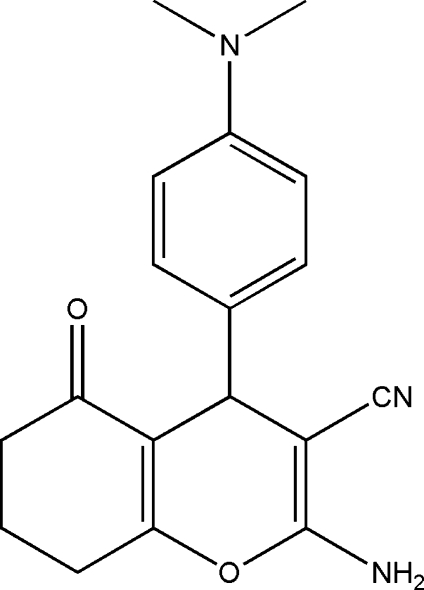


## Experimental

### 

#### Crystal data

C_18_H_19_N_3_O_2_
*M* *_r_* = 309.36
Monoclinic, 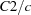

*a* = 25.021 (3) Å
*b* = 8.8724 (8) Å
*c* = 16.3827 (16) Å
β = 114.721 (2)°
*V* = 3303.5 (5) Å^3^
*Z* = 8
Mo *K*α radiation
μ = 0.08 mm^−1^
*T* = 298 K
0.40 × 0.36 × 0.22 mm

#### Data collection

Bruker SMART APEX CCD area-detector diffractometer
Absorption correction: multi-scan (*SADABS*; Sheldrick, 1996 ▶) *T*_min_ = 0.968, *T*_max_ = 0.982
8056 measured reflections
2907 independent reflections
1411 reflections with *I* > 2σ(*I*)
*R*_int_ = 0.062

#### Refinement

*R*[*F* ^2^ > 2σ(*F* ^2^)] = 0.060
*wR*(*F* ^2^) = 0.187
*S* = 1.01
2907 reflections
210 parameters
H-atom parameters constrained
Δρ_max_ = 0.24 e Å^−3^
Δρ_min_ = −0.21 e Å^−3^

### 

Data collection: *SMART* (Bruker, 2007 ▶); cell refinement: *SAINT* (Bruker, 2007 ▶); data reduction: *SAINT*; program(s) used to solve structure: *SHELXS97* (Sheldrick, 2008 ▶); program(s) used to refine structure: *SHELXL97* (Sheldrick, 2008 ▶); molecular graphics: *SHELXTL* (Sheldrick, 2008 ▶); software used to prepare material for publication: *SHELXTL*.

## Supplementary Material

Crystal structure: contains datablock(s) I, global. DOI: 10.1107/S1600536811043662/cv5176sup1.cif

Structure factors: contains datablock(s) I. DOI: 10.1107/S1600536811043662/cv5176Isup2.hkl

Supplementary material file. DOI: 10.1107/S1600536811043662/cv5176Isup3.cml

Additional supplementary materials:  crystallographic information; 3D view; checkCIF report

## Figures and Tables

**Table 1:** Hydrogen-bond geometry (Å, °)

*D*—H⋯*A*	*D*—H	H⋯*A*	*D*⋯*A*	*D*—H⋯*A*
N1—H1*A*⋯N2^i^	0.86	2.16	3.014 (4)	171
N1—H1*B*⋯O2^ii^	0.86	2.02	2.867 (4)	169

Symmetry codes: (i) 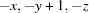; (ii) 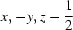.

**Fig. 1.**
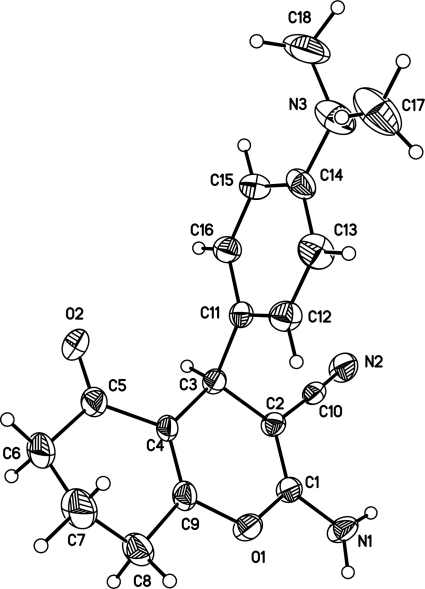
The title molecule with the atomic numbering scheme. The displacement ellipsoids are shown at the 30% probability level.

## supplementary crystallographic information

### Comment

In continuation of our structural studies of benzopyran derivatives (Kong *et al.*, 2011), we present here the title compound (I).

In (I) (Fig. 1), all bond lengths and angles are normal and comparable with those in close compounds (Kong *et al.*, 2011; Wang, 2011). The dihedral angle between the C2/C1/O1/C9/C4 plane and the (C2-C4) plane is 9.86( 4 )°. The C2/C1/O1/C9/C4 plane forms an angle of 86.43 (12 )° with the phenyl plane C11-C16.

In the crystal structure, intermolecular N—H···N and N—H···O hydrogen bonds (Table 1) link molecules into corrugated layers parallel to *bc* plane.

### Experimental

Malononitrile  (6 mmol), 1,3-cyclohexanedione (6 mmol) and N,N-dimethylbenzaldehyde (6 mmol) were dissolved in 20 ml ethanol ml in a round-bottom flask. The mixture was warmed, with agitation, to 363 K over a period of  5 h. The resulting solution was cooled. Crystals of (I) suitable for X-ray diffraction analysis were obtained by slow evaporation.

### Refinement

All H atoms were placed in geometrically idealized positions (N-H 0.86 and C-H 0.93-0.97 Å ) and treated as riding on their parent atoms, with U_iso_(H) = 1.2-1.5 U_eq_ (C, N).

### Crystal data

**Table:**

C_18_H_19_N_3_O_2_	*F*(000) = 1312
*M**_r_* = 309.36	*D*_x_ = 1.244 Mg m^−^^3^
Monoclinic, *C*2/*c*	Mo *K*α radiation, λ = 0.71073 Å
*a* = 25.021 (3) Å	Cell parameters from 1265 reflections
*b* = 8.8724 (8) Å	θ = 2.5–21.3°
*c* = 16.3827 (16) Å	µ = 0.08 mm^−^^1^
β = 114.721 (2)°	*T* = 298 K
*V* = 3303.5 (5) Å^3^	Block, red
*Z* = 8	0.40 × 0.36 × 0.22 mm

### Data collection

**Table:**

Bruker SMART APEX CCD area-detector diffractometer	2907 independent reflections
Radiation source: fine-focus sealed tube	1411 reflections with *I* > 2σ(*I*)
graphite	*R*_int_ = 0.062
phi and ω scans	θ_max_ = 25.0°, θ_min_ = 2.5°
Absorption correction: multi-scan (*SADABS*; Sheldrick, 1996)	*h* = −29→25
*T*_min_ = 0.968, *T*_max_ = 0.982	*k* = −10→8
8056 measured reflections	*l* = −19→19

### Refinement

**Table:**

Refinement on *F*^2^	Primary atom site location: structure-invariant direct methods
Least-squares matrix: full	Secondary atom site location: difference Fourier map
*R*[*F*^2^ > 2σ(*F*^2^)] = 0.060	Hydrogen site location: inferred from neighbouring sites
*wR*(*F*^2^) = 0.187	H-atom parameters constrained
*S* = 1.01	*w* = 1/[σ^2^(*F*_o_^2^) + (0.0818*P*)^2^ + 1.1159*P*] where *P* = (*F*_o_^2^ + 2*F*_c_^2^)/3
2907 reflections	(Δ/σ)_max_ < 0.001
210 parameters	Δρ_max_ = 0.24 e Å^−^^3^
0 restraints	Δρ_min_ = −0.21 e Å^−^^3^

### Special details

**Table:**

Geometry. All esds (except the esd in the dihedral angle between two l.s. planes) are estimated using the full covariance matrix. The cell esds are taken into account individually in the estimation of esds in distances, angles and torsion angles; correlations between esds in cell parameters are only used when they are defined by crystal symmetry. An approximate (isotropic) treatment of cell esds is used for estimating esds involving l.s. planes.
Refinement. Refinement of F^2^ against ALL reflections. The weighted R-factor wR and goodness of fit S are based on F^2^, conventional R-factors R are based on F, with F set to zero for negative F^2^. The threshold expression of F^2^ > 2sigma(F^2^) is used only for calculating R-factors(gt) etc. and is not relevant to the choice of reflections for refinement. R-factors based on F^2^ are statistically about twice as large as those based on F, and R- factors based on ALL data will be even larger.

### Fractional atomic coordinates and isotropic or equivalent isotropic displacement parameters (Å^2^)

**Table:**

	*x*	*y*	*z*	*U*_iso_*/*U*_eq_	
N1	0.05600 (14)	0.2235 (3)	−0.03033 (19)	0.0617 (9)	
H1A	0.0417	0.3129	−0.0433	0.074*	
H1B	0.0652	0.1742	−0.0679	0.074*	
N2	0.00475 (15)	0.4788 (4)	0.0962 (2)	0.0641 (10)	
N3	0.23314 (16)	0.5169 (4)	0.4808 (3)	0.0788 (11)	
O1	0.08809 (11)	0.0187 (2)	0.05519 (15)	0.0530 (7)	
O2	0.07367 (12)	−0.0804 (3)	0.32595 (18)	0.0683 (8)	
C1	0.06404 (14)	0.1610 (4)	0.0482 (2)	0.0422 (9)	
C2	0.05254 (14)	0.2195 (3)	0.1146 (2)	0.0389 (8)	
C3	0.06843 (14)	0.1431 (3)	0.2039 (2)	0.0383 (8)	
H3	0.0327	0.1385	0.2145	0.046*	
C4	0.08682 (14)	−0.0157 (3)	0.1978 (2)	0.0390 (8)	
C5	0.08851 (16)	−0.1218 (4)	0.2673 (3)	0.0543 (10)	
C6	0.1057 (2)	−0.2815 (4)	0.2617 (3)	0.0838 (15)	
H6A	0.0704	−0.3419	0.2335	0.101*	
H6B	0.1276	−0.3199	0.3220	0.101*	
C7	0.1428 (2)	−0.2992 (4)	0.2089 (3)	0.0866 (15)	
H7A	0.1814	−0.2557	0.2427	0.104*	
H7B	0.1479	−0.4055	0.2003	0.104*	
C8	0.11417 (18)	−0.2228 (4)	0.1188 (3)	0.0613 (11)	
H8A	0.1417	−0.2193	0.0911	0.074*	
H8B	0.0801	−0.2805	0.0799	0.074*	
C9	0.09573 (14)	−0.0674 (4)	0.1286 (2)	0.0449 (9)	
C10	0.02665 (16)	0.3637 (4)	0.1031 (2)	0.0428 (9)	
C11	0.11413 (14)	0.2331 (3)	0.2807 (2)	0.0386 (8)	
C12	0.16973 (16)	0.2606 (4)	0.2860 (3)	0.0555 (10)	
H12	0.1808	0.2171	0.2438	0.067*	
C13	0.20934 (17)	0.3510 (4)	0.3524 (3)	0.0633 (11)	
H13	0.2464	0.3673	0.3537	0.076*	
C14	0.19513 (17)	0.4180 (4)	0.4172 (3)	0.0522 (10)	
C15	0.13956 (18)	0.3880 (4)	0.4126 (2)	0.0572 (10)	
H15	0.1283	0.4297	0.4551	0.069*	
C16	0.10079 (15)	0.2973 (4)	0.3461 (2)	0.0490 (9)	
H16	0.0640	0.2788	0.3454	0.059*	
C17	0.2940 (2)	0.5246 (5)	0.4932 (4)	0.1074 (19)	
H17A	0.3129	0.4299	0.5158	0.161*	
H17B	0.3139	0.6030	0.5354	0.161*	
H17C	0.2953	0.5460	0.4367	0.161*	
C18	0.2225 (2)	0.5576 (6)	0.5575 (3)	0.1047 (18)	
H18A	0.1839	0.6007	0.5377	0.157*	
H18B	0.2513	0.6299	0.5932	0.157*	
H18C	0.2251	0.4694	0.5929	0.157*	

### Atomic displacement parameters (Å^2^)

**Table:**

	*U*^11^	*U*^22^	*U*^33^	*U*^12^	*U*^13^	*U*^23^
N1	0.104 (3)	0.0475 (19)	0.046 (2)	0.0274 (17)	0.0439 (19)	0.0138 (15)
N2	0.092 (3)	0.048 (2)	0.059 (2)	0.0198 (19)	0.038 (2)	0.0132 (16)
N3	0.069 (3)	0.064 (2)	0.079 (3)	−0.0101 (19)	0.006 (2)	−0.019 (2)
O1	0.0782 (18)	0.0426 (15)	0.0480 (16)	0.0211 (13)	0.0360 (14)	0.0096 (12)
O2	0.097 (2)	0.0645 (18)	0.0472 (17)	−0.0115 (15)	0.0338 (16)	0.0095 (14)
C1	0.050 (2)	0.038 (2)	0.040 (2)	0.0075 (16)	0.0202 (17)	0.0037 (16)
C2	0.045 (2)	0.037 (2)	0.0331 (19)	0.0050 (15)	0.0149 (16)	0.0038 (15)
C3	0.042 (2)	0.0386 (19)	0.037 (2)	0.0015 (15)	0.0187 (16)	0.0036 (15)
C4	0.047 (2)	0.0325 (19)	0.0336 (19)	−0.0056 (15)	0.0128 (16)	0.0051 (15)
C5	0.066 (3)	0.046 (2)	0.040 (2)	−0.0094 (19)	0.011 (2)	0.0049 (18)
C6	0.139 (4)	0.039 (2)	0.061 (3)	0.002 (2)	0.030 (3)	0.015 (2)
C7	0.120 (4)	0.046 (3)	0.081 (3)	0.025 (3)	0.029 (3)	0.016 (2)
C8	0.074 (3)	0.040 (2)	0.067 (3)	0.0051 (19)	0.027 (2)	0.0000 (19)
C9	0.052 (2)	0.035 (2)	0.045 (2)	0.0021 (16)	0.0178 (18)	0.0088 (17)
C10	0.060 (2)	0.039 (2)	0.033 (2)	0.0017 (18)	0.0232 (17)	0.0030 (16)
C11	0.044 (2)	0.0339 (19)	0.036 (2)	0.0044 (15)	0.0153 (16)	0.0061 (15)
C12	0.049 (2)	0.064 (3)	0.058 (3)	−0.001 (2)	0.026 (2)	−0.007 (2)
C13	0.042 (2)	0.070 (3)	0.074 (3)	−0.004 (2)	0.021 (2)	−0.008 (2)
C14	0.056 (3)	0.033 (2)	0.052 (2)	0.0002 (18)	0.008 (2)	0.0012 (18)
C15	0.073 (3)	0.051 (2)	0.051 (2)	−0.005 (2)	0.029 (2)	−0.0125 (19)
C16	0.049 (2)	0.052 (2)	0.048 (2)	−0.0072 (18)	0.0225 (19)	−0.0072 (18)
C17	0.062 (3)	0.083 (4)	0.132 (5)	−0.018 (3)	−0.003 (3)	−0.018 (3)
C18	0.124 (4)	0.085 (3)	0.072 (4)	−0.020 (3)	0.009 (3)	−0.037 (3)

### Geometric parameters (Å, °)

**Table:**

N1—C1	1.337 (4)	C7—C8	1.505 (5)
N1—H1A	0.8600	C7—H7A	0.9700
N1—H1B	0.8600	C7—H7B	0.9700
N2—C10	1.141 (4)	C8—C9	1.484 (5)
N3—C14	1.390 (5)	C8—H8A	0.9700
N3—C18	1.435 (6)	C8—H8B	0.9700
N3—C17	1.450 (5)	C11—C16	1.372 (4)
O1—C9	1.369 (4)	C11—C12	1.379 (5)
O1—C1	1.383 (4)	C12—C13	1.381 (5)
O2—C5	1.222 (4)	C12—H12	0.9300
C1—C2	1.341 (4)	C13—C14	1.387 (5)
C2—C10	1.411 (5)	C13—H13	0.9300
C2—C3	1.507 (4)	C14—C15	1.386 (5)
C3—C4	1.498 (4)	C15—C16	1.376 (5)
C3—C11	1.524 (4)	C15—H15	0.9300
C3—H3	0.9800	C16—H16	0.9300
C4—C9	1.326 (4)	C17—H17A	0.9600
C4—C5	1.464 (5)	C17—H17B	0.9600
C5—C6	1.495 (5)	C17—H17C	0.9600
C6—C7	1.517 (6)	C18—H18A	0.9600
C6—H6A	0.9700	C18—H18B	0.9600
C6—H6B	0.9700	C18—H18C	0.9600
			
C1—N1—H1A	120.0	C7—C8—H8A	109.5
C1—N1—H1B	120.0	C9—C8—H8B	109.5
H1A—N1—H1B	120.0	C7—C8—H8B	109.5
C14—N3—C18	119.7 (4)	H8A—C8—H8B	108.1
C14—N3—C17	118.9 (4)	C4—C9—O1	123.1 (3)
C18—N3—C17	115.7 (4)	C4—C9—C8	125.8 (3)
C9—O1—C1	118.6 (2)	O1—C9—C8	111.1 (3)
N1—C1—C2	128.6 (3)	N2—C10—C2	177.2 (4)
N1—C1—O1	110.1 (3)	C16—C11—C12	116.5 (3)
C2—C1—O1	121.3 (3)	C16—C11—C3	121.2 (3)
C1—C2—C10	119.0 (3)	C12—C11—C3	122.3 (3)
C1—C2—C3	123.7 (3)	C11—C12—C13	121.7 (3)
C10—C2—C3	117.3 (3)	C11—C12—H12	119.2
C4—C3—C2	108.8 (3)	C13—C12—H12	119.2
C4—C3—C11	113.7 (3)	C12—C13—C14	121.5 (4)
C2—C3—C11	111.5 (3)	C12—C13—H13	119.2
C4—C3—H3	107.5	C14—C13—H13	119.2
C2—C3—H3	107.5	C15—C14—C13	116.6 (3)
C11—C3—H3	107.5	C15—C14—N3	121.2 (4)
C9—C4—C5	118.8 (3)	C13—C14—N3	122.1 (4)
C9—C4—C3	123.3 (3)	C16—C15—C14	121.0 (3)
C5—C4—C3	117.6 (3)	C16—C15—H15	119.5
O2—C5—C4	119.9 (3)	C14—C15—H15	119.5
O2—C5—C6	121.6 (3)	C11—C16—C15	122.7 (3)
C4—C5—C6	118.5 (4)	C11—C16—H16	118.6
C5—C6—C7	113.2 (3)	C15—C16—H16	118.6
C5—C6—H6A	108.9	N3—C17—H17A	109.5
C7—C6—H6A	108.9	N3—C17—H17B	109.5
C5—C6—H6B	108.9	H17A—C17—H17B	109.5
C7—C6—H6B	108.9	N3—C17—H17C	109.5
H6A—C6—H6B	107.8	H17A—C17—H17C	109.5
C8—C7—C6	111.0 (4)	H17B—C17—H17C	109.5
C8—C7—H7A	109.4	N3—C18—H18A	109.5
C6—C7—H7A	109.4	N3—C18—H18B	109.5
C8—C7—H7B	109.4	H18A—C18—H18B	109.5
C6—C7—H7B	109.4	N3—C18—H18C	109.5
H7A—C7—H7B	108.0	H18A—C18—H18C	109.5
C9—C8—C7	110.8 (3)	H18B—C18—H18C	109.5
C9—C8—H8A	109.5		

### Hydrogen-bond geometry (Å, °)

**Table:**

*D*—H···*A*	*D*—H	H···*A*	*D*···*A*	*D*—H···*A*
N1—H1A···N2^i^	0.86	2.16	3.014 (4)	171.
N1—H1B···O2^ii^	0.86	2.02	2.867 (4)	169.

Symmetry codes: (i) −*x*, −*y*+1, −*z*; (ii) *x*, −*y*, *z*−1/2.
